# DCAF-GAN: Enhancing historical landscape restoration with dual-branch feature extraction and attention fusion

**DOI:** 10.1371/journal.pone.0334532

**Published:** 2025-10-29

**Authors:** Li Fang, Bo Han, Mingyan Bi, Lihui Wang, Dandan Wang

**Affiliations:** 1 College of Architecture and Civil Engineering, Qiqihar University, Qiqihar, China; 2 College of Computer and Control Engineering, Qiqihar University, Qiqihar, China; Nanchang University, CHINA

## Abstract

Historical landscape restoration has become a crucial area of research in cultural heritage preservation, and with the advancement of digital technologies, effectively restoring damaged historical images has become a critical challenge. Traditional restoration methods face difficulties in handling large occlusions, complex structural features, and maintaining high fidelity in restored images. Existing deep learning methods often focus on restoring a single feature, making it difficult to achieve high-quality reconstruction of both texture and structure. To address these challenges, we propose DCAF-GAN, a novel deep learning model that effectively restores both fine textures and global structures in damaged historical landscapes through a dual-branch encoder and a channel attention-guided fusion module. Experimental results show that DCAF-GAN achieves a PSNR of 29.12 and SSIM of 0.867 on the StreetView dataset, and a PSNR of 28.6 and SSIM of 0.854 on the Places2 dataset, significantly outperforming other models. These results demonstrate that DCAF-GAN not only provides high-quality restorations but also maintains computational efficiency. DCAF-GAN offers a promising solution for the digital preservation and restoration of cultural heritage, with significant potential for further applications.

## Introduction

Historical landscapes carry the memory of human civilization and are an important part of cultural heritage preservation and transmission. From ancient architecture, murals, and stone carvings to garden design and urban planning, these landscapes not only record the artistic styles and engineering techniques of specific eras but also embody information on social structures, religious beliefs, and ecological concepts [1,2]. However, due to prolonged exposure to natural environmental changes, climate impacts, war damage, and human intervention, many historical landscapes have suffered severe destruction or have even completely disappeared. For example, parts of the Forbidden City’s ancient buildings were burned down due to war, the murals of the Mogao Caves in Dunhuang have experienced peeling due to humidity changes, and many medieval castles in Europe have been damaged by weathering and urban expansion. The destruction of these historical landscapes not only signifies the loss of physical forms but also affects cultural continuity and the interpretation of historical information [3,4]. Therefore, how to use modern technology for the restoration of historical landscapes is not only a significant challenge in the field of cultural heritage preservation but also a key problem where computer vision and artificial intelligence technologies can play a role.

Traditional methods for historical landscape restoration mainly rely on archaeological research, documentary analysis, manual drawing, and computer-aided design (CAD) [5,6]. Archaeologists infer the original appearance of relics through cultural remains, historical documents, and image materials, while artists and architects use hand-drawing and three-dimensional modeling to conduct restoration. However, these methods have several limitations. First, the manual restoration process depends on the expertise of specialists, which introduces subjectivity and can lead to uncertainty in the restoration results due to missing data or misinterpretations [7–9]. Additionally, manual restoration typically requires substantial time and labor, making it difficult to apply to large-scale landscape restoration projects. With the development of computer technology, restoration methods based on image processing and 3D modeling have gradually emerged. However, such methods often rely on existing complete data for reconstruction, and when faced with large-scale damage or severe deterioration, the restoration results remain unsatisfactory [10–12]. Therefore, researching an efficient, intelligent, and scalable historical landscape restoration method to achieve more precise structural reconstruction and detailed repair holds significant academic value and application prospects.

In recent years, deep learning technology, especially Generative Adversarial Networks (GANs), has achieved breakthrough progress in the field of computer vision. GANs utilize adversarial training between a generator and a discriminator to learn data distributions and generate samples similar to real data. This technology has been widely applied to tasks such as image inpainting, super-resolution reconstruction, and style transfer [13]. For instance, DeepFillv2 employs generative networks to fill missing areas, SRGAN enhances the clarity of low-resolution images through adversarial learning, and SinGAN achieves multi-scale image synthesis through single-image learning [14–16]. However, in the specific task of historical landscape restoration, existing methods still struggle to restore fine textures while ensuring global consistency, leading to structural deviations or local blurring in the generated images. Furthermore, the role of the discriminator in adversarial training directly impacts the performance of the generator. Traditional CNN-based discriminators often struggle to extract global information when processing high-resolution, complex-textured historical landscape images, causing the generator to fail to effectively learn the overall structural distribution patterns [17–20]. Therefore, in historical landscape restoration, it is essential to design a more effective discriminator that not only captures fine-grained details but also enhances the generator’s ability to learn and preserve the global structural context, ultimately improving the restoration quality.

To overcome the challenges of restoring both fine textures and global consistency in historical landscapes, this paper proposes a dual-branch channel attention fusion generative adversarial network (DCAF-GAN) for high-quality historical landscape restoration and generation. Our method employs dual-branch feature extraction, where the texture encoder is responsible for extracting local details, and the structure encoder focuses on global contour information. Through channel attention-guided fusion (CAGF), the contributions of texture and structure features are dynamically adjusted to ensure that the restoration results have both clear details and overall consistency. Additionally, the discriminator adopts the ConvNeXt architecture to enhance the authenticity of generated images and improve the stability of adversarial training. Experimental results show that DCAF-GAN performs excellently on multiple historical landscape datasets, effectively restoring missing areas while maintaining consistency in historical styles.

The main contributions of this paper are as follows:

- This paper proposes a generative adversarial network (DCAF-GAN) that incorporates dual-branch feature extraction and channel attention-guided fusion, enabling high-quality historical landscape restoration and generation.
- This paper designs a CAGF mechanism that dynamically adjusts the weights of different feature branches through global pooling and channel attention, thereby enhancing the clarity and consistency of restoration results.
- This paper adopts ConvNeXt as the discriminator to optimize adversarial training, improve the authenticity of restored images, and enhance the overall stability of the model.

## Related work

### Traditional restoration methods

The restoration of historical landscapes and ancient artworks has long relied on traditional image restoration techniques, which primarily use rule-based methods to fill missing areas, remove scratches, repair damaged parts, and preserve the original artistic style as much as possible. Early restoration techniques mainly focused on interpolation-based methods, texture synthesis, and prior information-based approaches. These methods could restore the integrity of images to some extent but still had many limitations when dealing with complex damaged areas.

Interpolation-based methods were among the earliest techniques used for image restoration. They utilize information from surrounding pixels to fill damaged areas, minimizing visual discontinuities [21,22]. These methods are computationally efficient and perform well in cases of minor damage, but when applied to large missing areas, they tend to cause blurring or loss of local texture information. Additionally, gradient diffusion-based restoration methods attempt to propagate color and edge information within the image to fill missing areas, blending them with the surrounding environment [23–25]. However, these methods perform poorly when handling complex structures and struggle to accurately restore geometric details in images. To better preserve local textures in images, texture synthesis-based restoration methods were introduced. These methods analyze the texture features around damaged areas and generate texture structures matching the original image during the filling process, thereby improving the naturalness of restoration [26,27]. One representative method is patch-based inpainting, which synthesizes missing parts using small image patches. This approach is suitable for scenes with rich details and strong local repetition. However, when dealing with complex historical landscapes and irregularly shaped damaged areas, this method often fails to reconstruct missing information precisely, leading to inconsistencies or visual artifacts in the restored regions.

Another common category of restoration methods is probability model-based approaches, which use known image information to infer reasonable ways to fill missing parts. For example, some methods integrate distance fields to guide pixel filling, reducing structural distortion while maintaining smoothness in the restored areas [28,29]. Additionally, global optimization models have been applied to image restoration tasks by minimizing certain mathematical loss functions to ensure the continuity and rationality of the filled regions [30]. However, these methods often have high computational complexity in large-scale restoration tasks, making them difficult to apply to high-resolution historical landscape restoration. For different types of historical landscapes and artworks, traditional methods also vary accordingly. In the restoration of murals and calligraphy works, multi-layer structure segmentation methods are often employed to separately process overall structures and local details, making the restoration results more realistic [31–33]. In the restoration of traditional Chinese paintings, researchers have proposed damage-detection-based image restoration methods, where specific algorithms first identify damaged areas in the painting, followed by patch matching and texture synthesis to restore the image while maintaining stylistic consistency. Furthermore, to improve restoration accuracy, some methods incorporate spatial attention mechanisms to better balance local textures and overall layouts when dealing with large-scale damaged areas [34]. However, these methods still face adaptability issues when applied to highly complex painting styles and multi-layered artistic expressions.

Overall, traditional historical landscape restoration methods are effective for minor damage repair and regular texture filling but have limitations when dealing with large-scale damage, complex geometric structures, and highly artistic images. Additionally, restoration results from traditional methods often rely on manual parameter adjustments, making them unable to intelligently adapt to different types of historical landscapes. Therefore, exploring more efficient and intelligent restoration methods, particularly those integrating modern computer vision and deep learning technologies, has become a key research direction.

### Image restoration based on generative adversarial networks (GANs)

In recent years, Generative Adversarial Networks (GANs) have become an essential tool for image restoration and have been widely applied in the restoration of artworks and historical landscapes. Compared with traditional methods, GANs leverage adversarial training between a generator and a discriminator to effectively learn both the global structure and local textures of images, making the restoration results more natural and coherent. Many researchers have proposed various GAN-based restoration methods tailored to different types of image damage to enhance restoration quality and adaptability.

Some researchers have introduced a GAN restoration method based on context encoding, which employs conditional generation to infer missing content using surrounding pixel information [35]. This method performs well in repairing regularly shaped damaged areas. However, since a single discriminator struggles to simultaneously focus on both global layout and local details, another class of methods has adopted a global-local discriminator architecture [36–38]. In this approach, the global discriminator ensures overall stylistic consistency, while the local discriminator focuses on the realism of details within the restored areas, thereby improving the coherence and visual quality of the restoration results. Furthermore, to enhance semantic understanding during the restoration process, some scholars have incorporated attention mechanisms [39,40]. By leveraging self-attention modules, these models improve their ability to capture long-range dependencies, ensuring that the restored regions align better with the global style of the original image. For instance, some researchers have introduced channel attention and spatial attention modules into the GAN generator to more accurately focus on the features of damaged areas, thereby achieving clearer detail recovery [41]. For complex structural restoration tasks, some researchers have proposed edge-guided generative models. These methods first use an edge detection network to predict structural information in the missing regions and then employ GANs for detailed filling, ensuring structural integrity in the restored images [42–44]. Additionally, some studies have introduced multi-stage generation approaches, where a coarse restoration is performed first, followed by progressive refinement to enhance restoration quality in large missing areas and improve overall visual consistency. In the field of cultural heritage and traditional art restoration, some researchers have developed GAN models specifically designed for artistic styles. For example, some studies have combined GANs with style transfer techniques to ensure that the restored images not only fill missing parts but also retain the original artwork’s colors and brushstroke characteristics [45,46]. Another category of methods incorporates Variational Autoencoders (VAEs) and Transformer architectures, utilizing multi-scale feature extraction and cross-region information interaction to better preserve artistic styles and enhance the model’s generalization capability [47].

Overall, GANs have demonstrated high flexibility and superiority in image restoration tasks. Their effectiveness has been validated in numerous studies, particularly in the restoration of artworks and historical landscapes. As GAN architectures continue to be optimized—through the integration of attention mechanisms, multi-stage generation, and edge-guided methods—their applications in image restoration will become even more promising.

## Methods

### Overall framework

**Fig 1:**
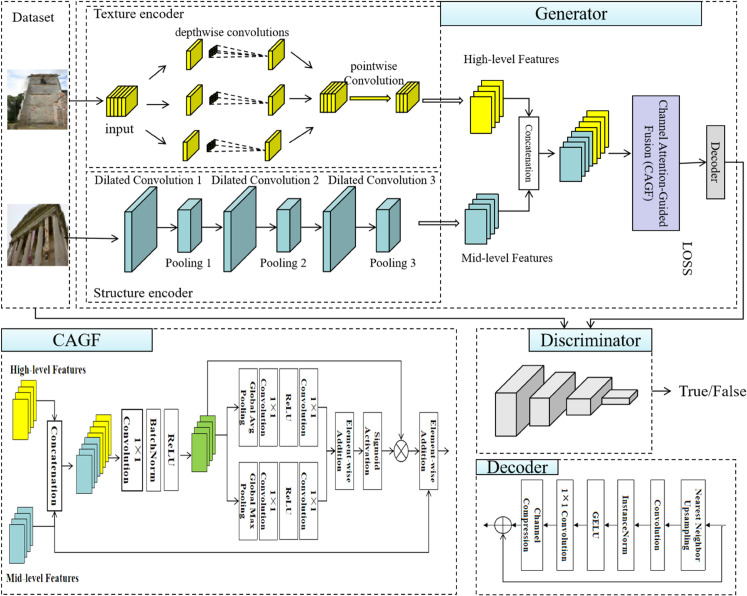
The overall architecture of the proposed DCAF-GAN model. The generator consists of a texture encoder and a structure encoder, the texture encoder includes depthwise and pointwise convolutions, and the structure encoder contains dilated convolutions and pooling operations. The CAGF module processes multi-scale features. The decoder comprises upsampling and residual connections. The discriminator is based on the ConvNeXt architecture.

The dual-branch channel attention fusion generative adversarial network (DCAF-GAN) proposed in this paper is designed to achieve high-quality historical landscape restoration. The overall framework consists of a generator and a discriminator, where the generator adopts a dual-branch structure to separately extract local texture information and global structural features. Through the channel attention fusion strategy, the complementary nature of different features is enhanced, ultimately generating high-quality restored images. The discriminator is built upon a deep convolutional structure based on ConvNeXt to improve the stability of adversarial training and optimize the learning capability of the generator. The architecture of the entire system is illustrated in Fig 1.

In the generator, a dual-branch encoder is designed to extract features separately for texture details and structural information. The texture encoder employs depthwise separable convolution (DW Conv) to efficiently extract local details, enhancing the texture expressiveness of the restored regions. Meanwhile, the structure encoder utilizes dilated convolution (Dilated Conv) to expand the receptive field, capturing global contour information and ensuring the rationality of the overall layout. The extracted features are then fed into the CAGF module, which dynamically adjusts the contribution of texture and structural features through global pooling (GPA) and channel-adaptive weighting (CA), ensuring that the restored regions maintain stylistic consistency with the original image. Finally, the fused features are reconstructed by the decoder, which integrates nearest-neighbor interpolation, residual blocks, and self-attention to enhance detail restoration and global consistency. The discriminator adopts the ConvNeXt architecture, which, compared to traditional CNN-based discriminators, can more effectively capture complex features in high-resolution images, improving the ability to assess the authenticity of the restored results. Through adversarial training, the discriminator continuously enhances the restoration quality of the generator, making the generated historical landscape images more realistic and coherent. The overall framework follows a multi-stage training strategy. First, the dual-branch encoder is trained independently to ensure that texture and structural features are effectively extracted. Next, the channel attention fusion module is trained to optimize the interaction between features. Finally, adversarial training is introduced to further enhance the restoration effect. The architectural design ensures both the clarity of detail restoration and the rationality of global structure, achieving high-quality historical landscape restoration.

### Generator

#### Dual-branch encoder and decoder.

The generator of DCAF-GAN adopts a dual-branch encoder structure, where the texture encoder focuses on extracting local high-frequency details to complete missing textures, while the structure encoder captures global contour information to ensure the overall consistency of the restored region. Given the input image *I*_*in*_, which consists of missing regions *M* and known areas, the dual-branch encoder extracts features separately, and the decoder reconstructs the final restored image *I*_*out*_.

**Dual-branch Encoder**

The dual-branch encoder consists of a texture encoder and a structure encoder, each learning different scale features to compensate for the limitations of a single-branch encoder. The texture encoder focuses on capturing fine-grained local features, while the structure encoder is responsible for preserving global structural information. The texture encoder employs depthwise separable convolution (DW Conv) to efficiently extract local features while reducing computational complexity. DW Conv decomposes standard convolution into depthwise convolution and pointwise convolution, allowing the model to efficiently capture local textures. After passing through *L* layers of DW Conv, the output of the texture encoder can be formulated as:

$$E_{t}=f_{t}(I_{in})=\sigma \left(W_{d}^{\left(L\right)}\;*\;\sigma \left(W_{d}^{\left(L-1\right)}\;*\;\cdots \sigma (W_{d}^{\left(1\right)}\;*\;I_{in}+b_{d}^{\left(1\right)})+\cdots \right)+b_{d}^{\left(L\right)}\right)$$ (1)

where $W_{d}^{\left(i\right)}$ and $b_{d}^{\left(i\right)}$ represent the convolution weights and biases of the *i*-th layer, * denotes the convolution operation, and σ(·) is the activation function (e.g., ReLU or GELU). This multi-layer convolutional design enables the network to effectively learn local patterns of missing areas and fill in missing textures.

The structure encoder utilizes dilated convolution (DC) to expand the receptive field and improve the ability to capture global structural information. The computation of dilated convolution is defined as:

$$E_{s}=f_{s}(I_{in})=\sigma \left(W_{d}^{\left(r_{L}\right)}*\sigma \left(W_{d}^{\left(r_{L-1}\right)}*\cdots \sigma (W_{d}^{\left(r_{1}\right)}*I_{in}+b_{d}^{\left(1\right)})+\cdots \right)+b_{d}^{\left(L\right)}\right)$$ (2)

where $W_{d}^{\left(r_{i}\right)}$ denotes the convolution kernel with dilation rate *r*_*i*_, and * represents the dilated convolution operation. When *r* > 1, empty spaces are inserted between convolution kernels, significantly expanding the receptive field:

Receptive Field=k+(k−1)(r−1) (3)

where *k* is the convolution kernel size, and *r* is the dilation rate. Dilated convolution allows the model to effectively restore global structure information, making the reconstructed region more natural. The final output of the dual-branch encoder is represented as:

$$E=[E_{t},E_{s}]$$ (4)

where *E*_*t*_ denotes texture features and *E*_*s*_ represents structural features. These features will be further refined in the CAGF module.

**Decoder**

The decoder maps the fused features back to the high-resolution image space and generates the final restored image *I*_*out*_. To ensure efficient reconstruction, the decoder adopts nearest neighbor upsampling and residual blocks for fine-grained detail enhancement.

To progressively restore image resolution, the decoder first performs upsampling using nearest neighbor interpolation:

$$E_{up}=U(E_{f})$$ (5)

where U(·) represents the upsampling operation, computed as:

$$I_{\text{up}}(x,y)=I\left(\lfloor x/s\rfloor ,\lfloor y/s\rfloor \right)$$ (6)

where *s* is the upsampling factor, and ⌊·⌋ denotes the floor operation. Nearest neighbor interpolation preserves edge information while reducing artifacts.

Following upsampling, the decoder employs multiple residual blocks to reconstruct fine-grained details. Each residual block is formulated as:

$$R(E)=E+\text{ReLU}(W_{1}\;*\;\text{ReLU}(W_{2}\;*\;E+b_{2})+b_{1})$$ (7)

where $W_{1},W_{2}$ are convolution weights, $b_{1},b_{2}$ are biases, and ReLU serves as the activation function. The residual connection stabilizes gradient propagation and enhances feature transfer. To further suppress artifacts and improve detail clarity, a 1×1 convolution is introduced for channel compression:

$$E_{\text{final}}=W_{f}\;*\;R(E_{up})+b_{f}$$ (8)

where *W*_*f*_ and *b*_*f*_ are the convolution weights and biases.

The final restored image *I*_*out*_ should be as close as possible to the ground truth image *I*_*gt*_, ensuring perceptual and structural consistency:

$$I_{out}=f_{dec}(E_{\text{final}}),\;\text{where}\;I_{gt}=\text{Ground Truth Image}$$ (9)

where $f_{dec}(\cdot )$ represents the decoder network, which integrates local and global features to achieve high-quality historical landscape restoration.

This section introduced the design of the dual-branch encoder and decoder, where the texture encoder employs depthwise separable convolution to enhance local feature extraction, while the structure encoder utilizes dilated convolution to capture global contour information. The decoder combines nearest neighbor upsampling and residual blocks to ensure the generated restored image retains fine details and structural coherence. In the next section, we will describe the CAGF module, which further optimizes feature interactions to produce more natural restoration results.

#### Channel attention-guided fusion (CAGF).

The generator of DCAF-GAN employs a dual-branch encoder to extract local texture information and global structural information. However, directly concatenating or summing these two types of features may lead to feature redundancy or imbalance, which can degrade the restoration quality. To address this issue, we introduce the CAGF module, which dynamically adjusts the contributions of different features. This ensures that the restored region retains overall structural consistency while recovering detailed texture information, allowing for better integration of features. The CAGF module integrates global feature statistics, adaptive channel weighting, and feature compression to refine the fusion process.

In the CAGF module, the output features from the dual-branch encoder, denoted as *f*_*hi*_ (high-level features) and *f*_*mi*_ (low-level features), are first concatenated along the channel dimension:

$$f_{t}=[f_{hi},f_{mi}]$$ (10)

where *f*_*hi*_ represents global structural information and *f*_*mi*_ represents local texture information. Since different regions of the image may require varying amounts of global and local information, a simple concatenation is not sufficient. To better balance the contributions of each feature type, a 1×1 convolution is applied to transform the feature representation:

$$f_{t}^{\prime }=\text{ReLU}(\text{BN}({\text{Conv}}_{1\;\times \;1}(f_{t})))$$ (11)

where ${\text{Conv}}_{1\;\times \;1}$ represents a 1×1 convolution operation, BN(·) denotes batch normalization, and ReLU(·) is the activation function. This operation normalizes the feature distribution before the attention mechanism is applied.

To further optimize feature selection, we employ a channel attention mechanism to adaptively adjust the importance of different channels. First, we extract global statistics from $f_{t}^{\prime }$ using global average pooling (GAP) and global max pooling (GMP):

$$C_{\mathrm{avg}}=\frac{1}{H\;\times \;W}\sum_{i=1}^{H}\sum_{j=1}^{W}f_{t}^{'}\left(i,\;j\right)$$ (12)

$$C_{\max}=\max_{i,j}\;f_{t}^{'}\left(i,\;j\right)$$ (13)

where *H* and *W* are the height and width of the feature map, respectively. $C_{\text{avg}}$ captures the overall feature distribution, while $C_{\text{max}}$ emphasizes the most significant activations. These two statistics are concatenated and passed through a multilayer perceptron (MLP) to compute channel-wise attention weights:

$$W_{c}=\sigma (W_{2}\delta (W_{1}[C_{\text{avg}},C_{\text{max}}]))$$ (14)

where *W*_1_ and *W*_2_ are weight matrices of the MLP, δ(·) represents the ReLU activation function, and σ(·) denotes the sigmoid function. This process allows the network to learn the relative importance of different feature channels. The computed attention weights are then applied to the feature map through element-wise multiplication:

$$f_{c}=W_{c}⊙f_{t}^{'}$$ (15)

where ⊙ denotes element-wise multiplication, ensuring that each channel is scaled according to its learned importance.

Finally, to preserve the original feature information, the attention-enhanced feature *f*_*c*_ is added back to the original transformed feature:

$$f_{\text{out}}=f_{c}+f_{t}^{'}$$ (16)

where $f_{\text{out}}$ is the final fused feature, which is fed into the decoder for reconstruction.

The CAGF module leverages channel attention to effectively regulate the contributions of different feature types. By dynamically adjusting feature weights, it enhances the model’s ability to focus on critical regions while suppressing redundant information. This fusion approach ensures that the restored image maintains structural coherence while preserving fine-grained texture details, improving the overall restoration quality.

### Discriminator

**Fig 2:**
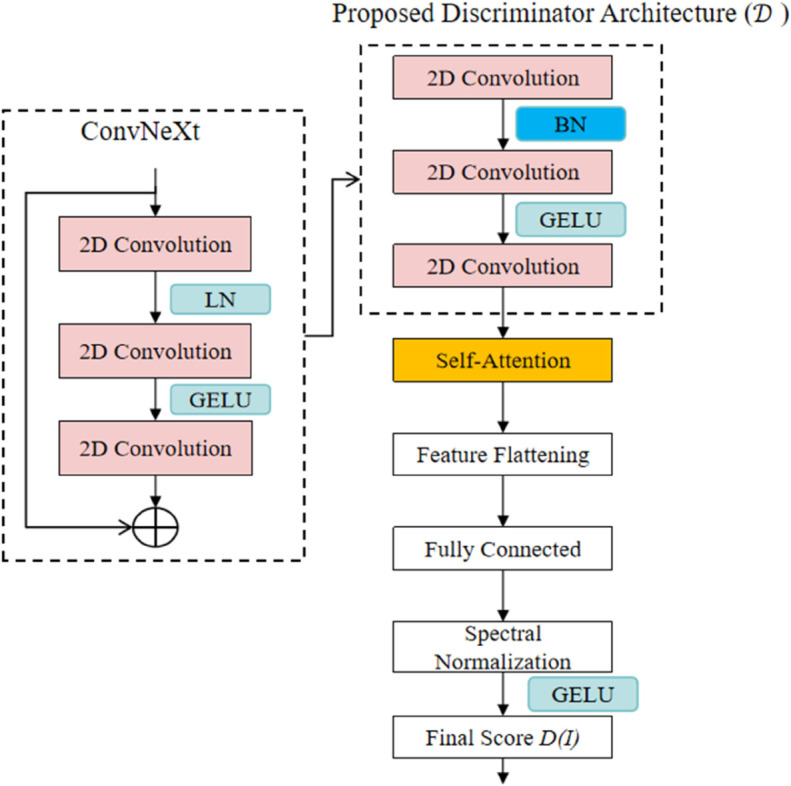
The proposed discriminator architecture consists of a ConvNeXt-based convolutional block, self-attention, and a fully connected layer with spectral normalization.

The discriminator in DCAF-GAN adopts the ConvNeXt architecture to enhance the stability of adversarial training and improve the ability to distinguish between real and generated images. ConvNeXt builds upon traditional convolutional neural networks (CNNs) by introducing deeper convolutional layers and efficient normalization techniques, demonstrating superior performance in image classification and generative tasks. To enable the discriminator to effectively distinguish between generated and real images, a multi-layer convolutional structure is designed to capture both local details and global patterns, thereby improving the evaluation accuracy of restoration quality, as shown in Fig 2.

The input to the discriminator consists of real images *I*_*gt*_ and generated images *I*_*out*_, with the objective of learning the distribution differences between the two. These images are processed through multiple ConvNeXt blocks for feature extraction. The output of the *l*-th convolutional layer, denoted as *f*_*l*_, is computed as:

$$f_{l}={\text{Conv}}_{l}(I)=\sigma (W_{l}\;*\;I+b_{l})$$ (17)

where *W*_*l*_ and *b*_*l*_ represent the convolution kernel weights and biases, * denotes the convolution operation, and σ(·) is the nonlinear activation function (e.g., GELU or ReLU). Through multiple layers of ConvNeXt blocks, the discriminator can capture features across different scales, ranging from low-level textures to high-level semantics, progressively enhancing its discrimination ability.

To further strengthen the global feature modeling capability of the discriminator, self-attention is incorporated into the ConvNeXt structure. The self-attention mechanism is formulated as:

$$\text{Attention}(Q,K,V)=\text{softmax}\left(\frac{QK^{T}}{\sqrt{d_{k}}}\right)V$$ (18)

where *Q*, *K*, and *V* represent the query, key, and value matrices, respectively, and *d*_*k*_ is the feature dimension. By computing relationships between different regions in the image, self-attention enhances the discriminator’s ability to capture global consistency, making it more effective in distinguishing between real and restored images, especially in complex damaged areas.

Additionally, to further stabilize the discriminator, Spectral Normalization is applied to constrain the Lipschitz constant of the model, thereby improving training stability. The spectral normalization is computed as:

$$W_{\text{SN}}=\frac{W}{\sigma_{\max}(W)}$$ (19)

where *W* represents the weight matrix and $\sigma_{\max}(W)$ is the largest singular value of *W*. This normalization technique prevents excessive optimization of the discriminator, enhances generalization, and contributes to more stable adversarial training.

Finally, the discriminator outputs a scalar *D*(*I*), representing the probability that the input image *I* is real. The discriminator is trained to minimize the binary cross-entropy loss function:

$$L_{D}=-{𝔼}_{I∼p_{\text{data}}}[\log D(I)]-{𝔼}_{I^{\prime }∼p_{\text{gen}}}[\log(1-D(I^{\prime }))]$$ (20)

where $p_{\text{data}}$ is the real image distribution, $p_{\text{gen}}$ is the generated image distribution, and *D*(*I*) is the discriminator’s output score.

In summary, incorporating the ConvNeXt structure allows the discriminator to effectively learn both local and global features while leveraging self-attention to improve discrimination in complex regions. Spectral normalization ensures stable training by preventing gradient explosion. This design ensures that the discriminator can effectively guide the generator during adversarial training, leading to improved realism in the restored images.

### Loss functions

The loss function design in DCAF-GAN aims to enhance the realism and structural consistency of restored images while ensuring training stability. To achieve this, we employ a combination of adversarial loss, perceptual loss, structural loss, and content loss, optimizing the generator’s performance to produce more visually authentic restorations.

The *adversarial loss* is the core component of the generative adversarial network (GAN), encouraging the generator to produce images indistinguishable from real ones. It is defined as follows:

$$L_{G}^{\text{adv}}=-{𝔼}_{I^{\prime }∼p_{\text{gen}}}[\log D(I^{\prime })]$$ (21)

$$L_{D}^{\text{adv}}=-{𝔼}_{I∼p_{\text{data}}}[\log D(I)]-{𝔼}_{I^{\prime }∼p_{\text{gen}}}[\log(1-D(I^{\prime }))]$$ (22)

where *D*(*I*) represents the discriminator’s probability of classifying input *I* as real, $p_{\text{data}}$ is the distribution of real images, and $p_{\text{gen}}$ represents the distribution of generated images. By optimizing this loss, the generator continuously improves the authenticity of restored images, while the discriminator learns to better distinguish between real and generated images.

The *perceptual loss* measures the similarity between generated and real images in high-level feature space. It extracts deep feature representations from a pre-trained VGG network and computes the Euclidean distance:

$$L_{p}=\sum_{l}{\left\|\phi_{l}(I_{\text{out}})-\phi_{l}(I_{\text{gt}})\right\|}_{2}$$ (23)

where $\phi_{l}(\cdot )$ denotes the feature map extracted from the *l*-th layer of the VGG network, and $I_{\text{out}}$ and $I_{\text{gt}}$ represent the generated and real images, respectively. This loss helps the generator capture fine details and texture information, improving visual quality.

The *structural loss* employs the Structural Similarity Index (SSIM) to evaluate the structural consistency between the restored and ground truth images:

$$L_{s}=1-SSIM(I_{\text{out}},I_{\text{gt}})$$ (24)

where SSIM(·) measures the similarity between two images based on luminance, contrast, and structure components. By minimizing this loss, the generator preserves structural coherence and reduces unwanted artifacts in the restored image.

The *content loss* directly computes the L1 pixel-wise difference between the generated and real images:

$$L_{c}={\left\|I_{\text{out}}-I_{\text{gt}}\right\|}_{1}$$ (25)

This loss ensures that the restored images maintain overall color consistency and brightness, preventing unrealistic artifacts or color distortions.

Finally, the total loss function for the generator combines these four losses to balance adversarial learning, visual fidelity, and structural consistency:

$$L_{G}=L_{G}^{\text{adv}}+\lambda_{1}L_{p}+\lambda_{2}L_{s}+\lambda_{3}L_{c}$$ (26)

where $\lambda_{1}$, $\lambda_{2}$, and $\lambda_{3}$ are weighting coefficients for the corresponding loss terms. By appropriately tuning these weights, DCAF-GAN maintains both the authenticity of restored images and the preservation of fine details, ensuring natural and coherent restoration results.

## Experiment

### Datasets

#### Dataset description.

This study employs the StreetView and Places2 datasets for training and testing to evaluate the effectiveness of DCAF-GAN in historical landscape restoration tasks.

The StreetView dataset primarily consists of urban street view images, including a large number of buildings, streets, and landmark landscapes. Some images contain occlusions, damage, or missing regions, making them suitable for simulating the restoration of historical buildings, streets, and cultural heritage sites. In our experiment, we select images featuring European-style architecture, historical districts, and classical landmarks to enable the model to learn the characteristic patterns of historical landscapes. The dataset also includes images with complex occlusions and intricate structural details, representing real-world challenges in historical restoration.

The Places2 dataset is a large-scale scene dataset covering over 400 categories and containing more than 18 million images. It is widely used in image generation, inpainting, and scene completion tasks. This dataset includes diverse categories such as natural landscapes, urban views, ancient buildings, and street scenes, allowing the model to learn a broader range of scene features and improve its generalization ability in different historical restoration tasks. Since Places2 contains a large number of landmark buildings, traditional streets, and cultural heritage sites, it plays a crucial role in enhancing the model’s restoration capabilities. The diversity of this dataset provides valuable insights into the model’s ability to handle a wide variety of restoration challenges, including urban and natural landscapes.

#### Preprocessing datasets.

Before training, all images are resized to a fixed resolution of 256 × 256 to meet the model’s input requirements. For high-resolution images in the Places2 dataset, random cropping is applied to extract 256 × 256 patches from the original large images, enhancing data diversity. Data augmentation techniques, including random horizontal flipping, color jittering, brightness adjustment, and contrast enhancement, are employed to improve the model’s generalization ability, making it more robust to variations in lighting conditions and viewpoints. To simulate real-world historical landscape degradation, artificial occlusions are introduced into the images. These occlusions simulate real-world historical degradation patterns, such as weathering and structural damage.

Rectangular occlusions are randomly generated across the images to simulate building damage and missing regions; irregular masking is applied to create randomly shaped occluded regions, mimicking natural deterioration and weathering effects; damage patterns derived from historical images are incorporated to provide realistic degradation scenarios, improving the model’s ability to adapt to real-world historical restoration tasks. During training, occlusion regions are randomly varied in size and position to ensure the model learns a wide range of restoration patterns, while in the testing phase, fixed masks are used to ensure fair comparison across different methods. These simulated degradations mimic real-world scenarios, such as color fading, weathering, and multi-layered occlusions, often seen in historical landscapes. To enhance data diversity, occlusion regions are designed to resemble real-world damage patterns, such as cracks and missing textures, often seen in historical landscapes.

Before being fed into the network, all images are normalized by scaling pixel values to the range [–1,1] to match the model’s computational requirements. After restoration, color matching techniques are applied to the repaired regions to align their appearance with the original image, ensuring visual consistency and improving the quality of the restored results.

### Hardware and software environment

This experiment is conducted on a high-performance computing platform to ensure the efficient training of DCAF-GAN and the generation of high-quality historical landscape restoration images. The hardware environment includes an NVIDIA A100 80GB or RTX 3090 24GB GPU, providing strong computational power, along with an Intel Xeon Gold 6226R @ 2.90GHz CPU and 256GB DDR4 memory to support large-scale data loading and model training. A 2TB NVMe SSD is used for high-speed data reading and writing. The software environment is based on the Ubuntu 20.04 LTS operating system, utilizing PyTorch 2.0 as the deep learning framework, with CUDA 11.8 and cuDNN 8.6 for GPU acceleration. OpenCV is used for image processing, NumPy and Matplotlib for data analysis, torchvision for dataset management, and tensorboard for training visualization. This configuration ensures efficient model training and the reproducibility of experiments.

### Training parameters and optimization strategy

The training of DCAF-GAN adopts the Adam optimizer with an initial learning rate of 2e-4, which is dynamically adjusted using cosine annealing scheduling to optimize model convergence speed and stability. The batch size is set to 16, and the total number of training epochs is 200, ensuring that the model sufficiently learns restoration patterns and generates high-quality historical landscape reconstructions.

The training process is divided into two stages. In the first stage, the generator is trained to learn the fundamental ability to fill missing regions, with L1 loss and SSIM loss being the primary optimization objectives to ensure structural consistency. In the second stage, the discriminator is introduced for adversarial training, increasing the weights of perceptual loss and adversarial loss to enhance the realism and detail of the restored images. To stabilize training, the discriminator employs Spectral Normalization to prevent gradient explosion or vanishing. The overall training process is outlined in Algorithm 1.

**Algorithm 1** Training procedure of DCAF-GAN

1: Initialize Generator *G*, Discriminator *D* with Spectral

Normalization

2: **for** each epoch *e* in total epochs **do**

3:   **for** each batch in dataset **do**

4:    Sample masked images *I*_*in*_ and ground truth images *I*_*gt*_

5:    Generate restored images $I_{out}=G(I_{in})$

6:    **if** *e* < 100 **then**

7:     Compute L1 content loss: $L_{c}=||I_{out}-I_{gt}|{|}_{1}$

8:     Compute structural loss: $L_{s}=1-SSIM(I_{out},I_{gt})$

9:     Compute total loss: $L_{G}=\lambda_{2}L_{s}+\lambda_{3}L_{c}$

10:    **else**

11:     Compute L1 content loss: $L_{c}=||I_{out}-I_{gt}|{|}_{1}$

12:     Compute structural loss: $L_{s}=1-SSIM(I_{out},I_{gt})$

13:     Compute perceptual loss: $L_{p}=||\phi (I_{out})-\phi (I_{gt})|{|}_{2}$

14:     Compute adversarial loss: $L_{adv}=-\log D(I_{out})$

15:     Compute total loss: $L_{G}=L_{adv}+\lambda_{1}L_{p}+\lambda_{2}L_{s}+\lambda_{3}L_{c}$

16:    **end if**

17:    Update generator *G* using Adam optimizer

18:    Compute discriminator loss: $L_{D}=-𝔼[\log D(I_{gt})]-𝔼[\log(1-D(I_{out}))]$

19:    Update discriminator *D* using Adam optimizer

20:   **end for**

21:   Adjust learning rate using cosine annealing

22: **end for**

The discriminator adopts Spectral Normalization to stabilize training, preventing gradient explosion or vanishing. During the early stage of training, L1 and SSIM losses dominate to ensure fundamental structural restoration. In the later stages, perceptual loss (VGG) and adversarial loss are incorporated to enhance texture realism and fine details. This approach enables DCAF-GAN to generate high-fidelity historical landscape restorations with both global consistency and fine-grained textures.

### Evaluation metrics

To comprehensively evaluate the performance of DCAF-GAN in historical landscape restoration tasks, several quantitative metrics are employed to assess the quality, structural consistency, and perceptual realism of the restored images.

First, the Structural Similarity Index (SSIM) is used to measure the similarity between the restored and ground truth images in terms of structural information. The SSIM value ranges from 0 to 1, where a higher value indicates better structural preservation. Peak Signal-to-Noise Ratio (PSNR) is also employed to evaluate image quality, with a higher PSNR value indicating lower distortion and better restoration fidelity. In addition, the Learned Perceptual Image Patch Similarity (LPIPS) metric is utilized, which is based on deep neural networks such as AlexNet or VGG to compute perceptual similarity in feature space. A lower LPIPS value suggests that the restored image is more visually similar to the ground truth. Furthermore, the Frechet Inception Distance (FID) is introduced to measure the distributional difference between generated and real images by computing the feature statistics from an Inception network. A lower FID score indicates that the restored images are closer to the real image distribution.

Additionally, to complement the quantitative metrics, a user perceptual study was conducted to evaluate the visual quality of the restored images from a human perspective. A group of 30 ordinary users was asked to rate the restored images based on clarity, color consistency, structural consistency, and visual appeal. These subjective ratings offer valuable insights into the visual realism of the restored images and help assess their suitability for cultural heritage restoration tasks. The results from this user study provide an additional layer of validation, ensuring that the model’s performance aligns with human visual perception.

The ratings were based on a five-point scale, as outlined in Table 1.

**Table 1:** Perceptual rating scale for user study.

Score	Criteria
Clarity	
1	The image is extremely blurry, with details almost impossible to identify.
2	The image is blurry, with insufficient detail restoration.
3	The image is slightly blurry, details are recognizable but not clear.
4	The image is clear, with good detail restoration.
5	The image is extremely clear, with precise details, perfectly restored.
Color Consistency	
1	The restored colors differ significantly from the original, with severe distortion.
2	The colors are different from the original, with noticeable color deviations.
3	The colors are close to the original, but there are deviations in some areas.
4	The colors are fairly consistent, with good restoration results.
5	The colors match the original perfectly, with natural and uniform tones.
Structural Consistency	
1	The structure is lost or severely distorted, the restoration fails to maintain the original structure.
2	The structure is somewhat distorted, partial restoration is poor.
3	The structure is partially restored, but some regions lose structure.
4	The structure is well-preserved, details are restored, though some areas can still be improved.
5	The structure is perfectly restored, consistent with the original.
Visual Appeal	
1	The image is unpleasant, with very poor restoration results.
2	The visual effect is poor, the restored image fails to provide a pleasant experience.
3	The visual effect is average, the image restoration is acceptable but not ideal.
4	The visual effect is good, the image restoration is natural.
5	The visual effect is excellent, the restored image is very natural and visually appealing.

These evaluation metrics comprehensively assess the performance of the model from multiple perspectives, ensuring that the restored images achieve both high visual quality and structural accuracy.

## Results

### Quantitative comparison analysis

To evaluate the effectiveness of DCAF-GAN in historical landscape restoration, we compare its performance with several state-of-the-art image restoration and inpainting models. The selected models include traditional Chinese painting restoration networks, super-resolution generative adversarial networks (GANs), and advanced image inpainting methods. Below is a brief introduction to each of the competing models:

SGRGAN (Sketch-Guided Restoration GAN) [48]: A restoration framework that utilizes sketch images as structural priors to restore traditional Chinese paintings while maintaining their unique artistic characteristics. It incorporates a Focal Block and BiSCCFormer Block to enhance the fusion of textural and structural features.

ConvSRGAN (Super-Resolution GAN for Chinese Paintings) [49]: A model designed for the super-resolution restoration of traditional Chinese landscape paintings. It employs an Enhanced Adaptive Residual Module and an Adaptive Deep Convolution Block to retain high-frequency details while preserving the painting’s artistic nuances.

NeXtSRGAN (ConvNeXt-based Super-Resolution GAN) [50]: A super-resolution model that integrates a ConvNeXt-based discriminator to improve realism and perceptual quality. It prioritizes visual fidelity over PSNR optimization, making it more suitable for complex restoration tasks.

EdgeConnect (Adversarial Edge Learning for Image Inpainting) [51]: A two-stage generative model that first predicts the missing edges using an edge generator, then reconstructs the missing regions via an image completion network, ensuring structural consistency.

DLP-GAN (Draw Modern Chinese Landscape Photos GAN) [52]: An unsupervised cross-domain image translation framework that translates traditional landscape paintings into modern photo-realistic images. It utilizes a dense-fusion module and asymmetric cycle mapping to balance realism and abstraction.

RestoreNet-Plus (Deep Learning-Based Optical Image Restoration) [53]: A deep-learning-based synthetic aperture imaging restoration network designed for optical imaging systems. It focuses on mitigating imaging blur, turbulence aberration, and noise to enhance restoration quality.

Table 2 presents the quantitative comparison between DCAF-GAN and other state-of-the-art models on the datasets. On the StreetView dataset, DCAF-GAN achieves a PSNR of 29.12 dB, significantly surpassing other methods, indicating its superior ability to restore image details while reducing noise. The SSIM score of 0.867 demonstrates that DCAF-GAN better preserves structural information compared to NeXtSRGAN (0.852) and RestoreNet-Plus (0.846). In terms of perceptual similarity, DCAF-GAN achieves an LPIPS score of 0.137, which is lower than EdgeConnect (0.158) and DLP-GAN (0.168), suggesting that the restored images are visually closer to the ground truth. Additionally, the FID value of 14.4 is significantly lower than other methods, indicating that the generated images exhibit higher overall quality and are more naturally aligned with real-world distributions. On the Places2 dataset, DCAF-GAN again demonstrates superior performance, achieving a PSNR of 28.6 dB, outperforming NeXtSRGAN (27.45 dB) and RestoreNet-Plus (26.95 dB), confirming its effectiveness in large-scale, complex scenes. The SSIM score of 0.854 further validates DCAF-GAN’s ability to maintain structural consistency across various damage types. In terms of perceptual similarity, DCAF-GAN achieves an LPIPS score of 0.143, lower than ConvSRGAN (0.176) and EdgeConnect (0.170), highlighting its capability in restoring fine details while ensuring high visual quality. The FID value of 15.3, the lowest among all methods, suggests that the restored images by DCAF-GAN maintain global coherence and closely resemble real-world image distributions. However, DCAF-GAN’s performance on larger occlusions and highly complex scenes may still be limited, as some blurring and artifacts remain in those regions, particularly in multi-layered and highly intricate structures. Overall, DCAF-GAN outperforms both datasets in PSNR, SSIM, LPIPS, and FID, demonstrating its effectiveness in historical landscape restoration. Its superior performance is attributed to the dual-branch encoder that captures both local textures and global structures, and the CAGF module, which balances feature types. Additionally, the optimized generator and discriminator architectures ensure high-quality restoration. The significant improvement in FID indicates better global consistency and perceptual realism. However, handling larger-scale occlusions and more complex scenarios remains a future challenge.

**Table 2:** Quantitative comparison on StreetView and Places2 datasets.

Model	StreetView				Places2			
	PSNR ↑	SSIM ↑	LPIPS ↓	FID ↓	PSNR ↑	SSIM ↑	LPIPS ↓	FID ↓
SGRGAN	26.45	0.834	0.172	19.1	25.78	0.819	0.185	20.4
ConvSRGAN	27.1	0.841	0.165	17.7	26.5	0.826	0.176	19
NeXtSRGAN	28.32	0.852	0.149	16.5	27.45	0.838	0.162	17.6
EdgeConnect	27.88	0.847	0.158	17.2	27.1	0.832	0.17	18.4
DLP-GAN	26.98	0.839	0.168	18.2	26.28	0.824	0.179	18.8
RestoreNet-Plus	27.45	0.846	0.155	16.3	26.95	0.83	0.167	17.3
DCAF-GAN (Ours)	29.12	0.867	0.137	14.4	28.6	0.854	0.143	15.3

**Fig 3:**
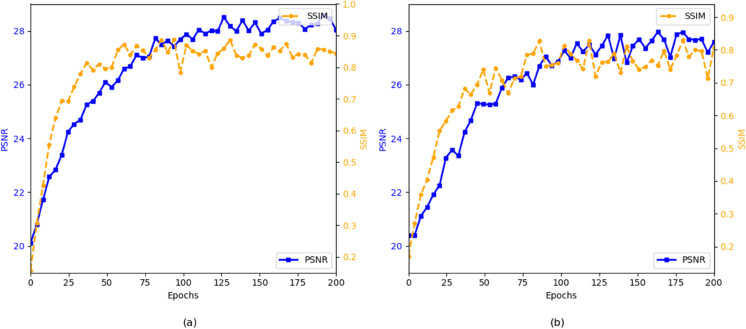
PSNR and SSIM vs Epoch for DCAF-GAN during training. (a) shows the performance on the StreetView dataset, and (b) shows the performance on the Places2 dataset.

The experimental convergence curves, as shown in the Fig 3 illustrate the training process of DCAF-GAN on different datasets, including the variation trends of PSNR (Peak Signal-to-Noise Ratio) and SSIM (Structural Similarity Index) over training epochs. From the figure, it is evident that the model converges rapidly in the early stages and gradually stabilizes, with noticeable differences in performance across datasets. Firstly, in subfigure (a), the training process of DCAF-GAN on the StreetView dataset is depicted. The PSNR curve starts from approximately 20, gradually increasing and stabilizing at around 28.7 after 150 epochs, indicating that the model progressively learns high-frequency details and improves restoration quality. Regarding SSIM, significant fluctuations are observed in the initial phase (first 50 epochs), reflecting instability in learning structural similarity. However, after 100 epochs, SSIM steadily increases and stabilizes at 0.95, demonstrating the model’s capability in restoring structural information effectively. Furthermore, the curves remain stable in the later training stages, suggesting that adversarial training (GAN) has reached an optimal generative effect on this dataset. In contrast, subfigure (b) presents the convergence behavior of DCAF-GAN on the Places2 dataset, where the performance is slightly inferior. The PSNR curve starts from approximately 20, gradually increasing to 28.2, which is slightly lower than that of the StreetView dataset. This suggests that the restoration task on this dataset is more challenging, particularly due to the increased scene complexity, including more occlusions and intricate details. Similarly, SSIM exhibits larger initial fluctuations and stabilizes at around 0.92 after 100 epochs, which is lower than that observed in the StreetView dataset. This implies that the model struggles more to restore globally consistent structures in Places2, possibly due to the dataset’s higher variability and more complex textures. From an overall perspective, the training curves of DCAF-GAN on both datasets display typical GAN training characteristics, where significant initial oscillations gradually stabilize. As adversarial training requires balancing the generator and discriminator, substantial fluctuations occur in the early training stages. However, thanks to the dual-branch CAGF and ConvNeXt-based discriminator, DCAF-GAN is able to stabilize training relatively quickly, entering a steady phase after 150 epochs while maintaining high PSNR and SSIM values.

**Fig 4:**
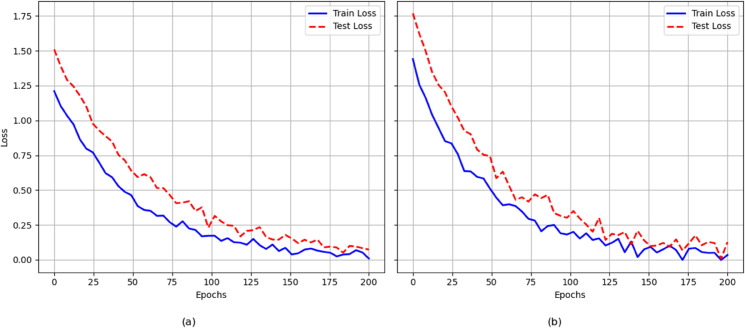
The training and testing loss curves for two datasets over 200 epochs.

The experimental loss curves in the Fig 4 illustrate the training and testing loss trends of DCAF-GAN on the datasets. From the curves, it can be observed that the model experiences a rapid decrease in loss during the initial training phase, indicating its ability to quickly learn fundamental features. As training progresses, the loss gradually decreases and stabilizes, suggesting that the model has reached convergence. On the StreetView dataset, both training and testing losses are relatively low, and the gap between them is small, indicating strong generalization performance with minimal overfitting. In contrast, on the Places2 dataset, the testing loss is slightly higher than the training loss, and the gap between them is more pronounced. This is likely due to the increased complexity of the dataset, making it more challenging for the model to generalize well to unseen samples. Overall, DCAF-GAN achieves stable convergence on both datasets, and despite the increased difficulty in more complex scenarios, it maintains a favorable training trend, demonstrating its adaptability.

### Computational cost analysis

Table 3 presents the computational cost comparison of DCAF-GAN with several benchmark models on the datasets. The results demonstrate that DCAF-GAN achieves significant advantages in computational efficiency and inference speed while maintaining a low parameter count and computational complexity. In terms of parameters, DCAF-GAN has only 24.4M (StreetView) and 24.6M (Places2), which is significantly lower than SGRGAN (85.7M/86.1M) and NeXtSRGAN (92.5M/93.0M), reducing the storage requirement by nearly 3-4 times. Compared to lighter models like EdgeConnect (64.3M/65.0M) and DLP-GAN (58.2M/59.0M), DCAF-GAN still exhibits a notable advantage, indicating that the model has undergone effective parameter optimization to minimize redundancy while maintaining high restoration quality. Regarding computational complexity, DCAF-GAN achieves only 19.7G FLOPs on the StreetView dataset and 19.9G FLOPs on the Places2 dataset. This represents more than a 50% reduction in computational cost compared to SGRGAN (42.3G/42.8G) and NeXtSRGAN (45.6G/46.2G). Even when compared to ConvSRGAN (38.1G/38.5G) and EdgeConnect (30.9G/31.5G), DCAF-GAN maintains the lowest computational cost, demonstrating its ability to process high-resolution images efficiently. These results suggest that DCAF-GAN is well-suited for large-scale data processing and real-time applications. For inference time, DCAF-GAN requires only 28.4ms on StreetView and 30.2ms on Places2, significantly faster than SGRGAN (92.5ms/95.2ms) and NeXtSRGAN (105.7ms/108.6ms), reducing the computation time by nearly three times. Even compared to more computationally efficient models like EdgeConnect (54.2ms/56.4ms) and RestoreNet-Plus (38.6ms/40.0ms), DCAF-GAN still maintains a faster inference speed. This is primarily due to the optimization of the dual-branch encoder and the channel attention-guided fusion module, which enhance computational efficiency when handling high-resolution images. Additionally, the use of a lightweight ConvNeXt-based discriminator reduces computational overhead, enabling high-quality restoration with a significantly lower inference cost. Overall, DCAF-GAN outperforms existing models in terms of parameters, computational complexity, and inference time, achieving a more lightweight and efficient design. These advantages make DCAF-GAN an optimal choice for historical landscape restoration and other applications requiring high-quality image restoration while maintaining computational efficiency. Particularly in tasks such as cultural heritage restoration and digital preservation, DCAF-GAN enables precise and efficient restoration with limited computational resources, enhancing its practicality in real-world applications.

**Table 3:** Computational cost comparison on StreetView and Places2 datasets.

Model	StreetView			Places2		
	Params (M) ↓	FLOPs (G) ↓	Inference Time (ms) ↓	Params (M) ↓	FLOPs (G) ↓	Inference Time (ms) ↓
SGRGAN	85.7	42.3	92.5	86.1	42.8	95.2
ConvSRGAN	76.4	38.1	78.3	76.9	38.5	80.1
NeXtSRGAN	92.5	45.6	105.7	93.0	46.2	108.6
EdgeConnect	64.3	30.9	54.2	65.0	31.5	56.4
DLP-GAN	58.2	27.8	43.1	59.0	28.2	45.3
RestoreNet-Plus	50.1	24.5	38.6	51.0	25.1	40.0
DCAF-GAN (Ours)	24.4	19.7	28.4	24.6	19.9	30.2

### Ablation study results and analysis

The ablation study aims to evaluate the contribution of each key module in DCAF-GAN to the historical landscape restoration task. Table 4 presents the results by removing different modules and conducting experiments on the dataset, changes in model performance can be observed. When the dual-branch encoder is removed, PSNR and SSIM significantly decrease, while LPIPS and FID increase, indicating that the absence of independent texture and structure encoding pathways severely affects the restoration capability. The PSNR on the StreetView dataset drops from 29.12 to 24.92, and on the Places2 dataset, it drops from 28.60 to 24.75. This demonstrates that the model struggles to effectively learn high-quality local details and global contours, leading to restoration results that deviate from the ground truth. The increase in FID further indicates a greater discrepancy between the generated images and real image distributions, resulting in unnatural image styles. These results confirm that the dual-branch encoder plays a crucial role in DCAF-GAN by separately extracting local texture information and global structural features, ensuring that the restored region aligns well with the overall scene. Further analysis of the texture encoder and structure encoder shows that removing the texture encoder leads to a slight decrease in PSNR and SSIM, but a more significant increase in LPIPS, suggesting that the absence of this module results in degraded texture quality in the restored regions, making local details appear blurry while maintaining overall contours. The LPIPS value on the Places2 dataset increases from 0.143 to 0.188, and on the StreetView dataset, it rises from 0.137 to 0.182, indicating a noticeable decline in perceptual quality. Removing the structure encoder results in a similar drop in PSNR, but a more significant decrease in SSIM, indicating that this module plays a crucial role in preserving the overall structure of the image. Comparatively, removing the texture encoder causes a greater increase in LPIPS, while removing the structure encoder leads to a more pronounced drop in SSIM. This further validates their complementary roles in DCAF-GAN, with the texture encoder focusing on local details and the structure encoder maintaining global layout consistency. When the channel attention-guided fusion module is removed, PSNR and SSIM decrease, while LPIPS and FID increase significantly. The FID value on the StreetView dataset increases from 14.4 to 17.8, and on the Places2 dataset, it increases from 15.3 to 18.9, indicating that the restored regions become less stylistically coherent with the original image. The channel attention-guided fusion module dynamically adjusts the contribution of texture and structure features, allowing the restored region to better match the style of the original image. Without this module, restored areas may appear inconsistent in style, with noticeable edges or mismatched colors. Finally, when the ConvNeXt-based discriminator is removed, PSNR remains relatively high, but FID deteriorates significantly. The FID value on the StreetView dataset increases from 14.4 to 16.5, and on the Places2 dataset, it increases from 15.3 to 17.1, indicating that the restored images deviate more from real image distributions. Although PSNR is not significantly affected, the absence of the discriminator reduces the visual consistency of the generated images, making the restorations appear less natural and realistic. The ConvNeXt-based discriminator, compared to traditional CNN discriminators, possesses a stronger feature extraction capability, allowing it to more effectively distinguish between real and generated images. This enhances the training of the generator and improves the realism of the restoration results. Consequently, removing this module does not significantly impact pixel-level losses but degrades overall perceptual quality and visual authenticity. Each module in DCAF-GAN is essential for historical landscape restoration. The dual-branch encoder ensures effective texture and structure extraction, with its removal causing the most significant drop in performance. The texture and structure encoders complement each other, while the channel attention-guided fusion module enhances feature interaction. The ConvNeXt-based discriminator improves perceptual quality, making restorations more realistic. Together, these components enable high-quality restoration, demonstrating DCAF-GAN’s potential in cultural heritage preservation.

**Table 4:** Ablation study results on StreetView and Places2 datasets.

Model Variant	StreetView				Places2			
	PSNR ↑	SSIM ↑	LPIPS ↓	FID ↓	PSNR ↑	SSIM ↑	LPIPS ↓	FID ↓
w/o Dual-branch Encoder	24.92	0.810	0.195	24.5	24.75	0.805	0.200	25.2
w/o Texture Encoder	25.80	0.822	0.182	22.3	25.50	0.818	0.188	23.1
w/o Structure Encoder	25.45	0.818	0.186	22.9	25.20	0.812	0.191	23.8
w/o Dual-branch Encoder + CAGF	26.95	0.837	0.165	19.7	26.65	0.830	0.171	20.4
w/o CAGF	27.68	0.846	0.153	17.8	27.30	0.841	0.160	18.9
w/o ConvNeXt Discriminator	28.20	0.852	0.145	16.5	27.85	0.847	0.150	17.1
Full Model (DCAF-GAN)	29.12	0.867	0.137	14.4	28.60	0.854	0.143	15.3

**Fig 5:**
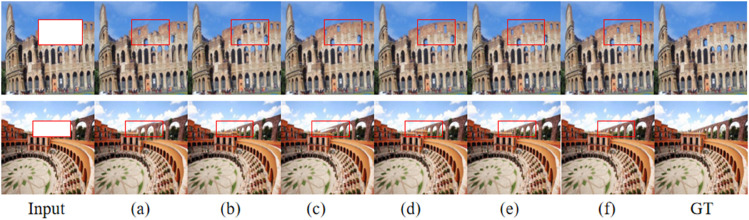
Qualitative comparison of restoration results under different loss weight settings. The input corrupted images are shown on the left, and the ground truth (GT) images are shown on the right. (a)-(f) represent the results obtained using different combinations of perceptual loss ($\lambda_{1}$), structural loss ($\lambda_{2}$), and content loss ($\lambda_{3}$). The specific weight settings are as follows: (a) $\lambda_{1}=1.0,\lambda_{2}=0.5,\lambda_{3}=0.5$ (perceptual loss dominant), (b) $\lambda_{1}=0.5,\lambda_{2}=1.0,\lambda_{3}=0.5$ (structural loss dominant), (c) $\lambda_{1}=0.5,\lambda_{2}=0.5,\lambda_{3}=1.0$ (content loss dominant), (d) $\lambda_{1}=1.0,\lambda_{2}=1.0,\lambda_{3}=0.5$ (balanced perceptual and structural losses), (e) $\lambda_{1}=1.0,\lambda_{2}=0.5,\lambda_{3}=1.0$ (balanced perceptual and content losses), (f) $\lambda_{1}=1.0,\lambda_{2}=1.0,\lambda_{3}=1.0$ (full model with all loss terms equally weighted).

The above Fig 5 illustrates the restoration performance of DCAF-GAN under different loss weight combinations, evaluating the impact of each loss term on the final generated results. The leftmost column presents the input corrupted images, while the rightmost column shows the corresponding ground truth (GT). The intermediate subfigures (a)-(f) represent the results obtained with different settings of perceptual loss ($\lambda_{1}$), structural loss ($\lambda_{2}$), and content loss ($\lambda_{3}$). The comparison reveals that different loss weight combinations significantly affect the quality of the restored images. In (a), where perceptual loss dominates, the generated images exhibit rich textures and details, but minor structural inconsistencies may be observed. In (b), with a dominant structural loss, the restored images maintain a stable overall shape and contour, though some finer texture details may be missing. In (c), with content loss being the primary factor, the images show strong pixel-level consistency in color but tend to appear overly smooth, lacking realism. In contrast, (d) and (e) demonstrate a balanced integration of perceptual and structural or content loss, resulting in a better trade-off between structural integrity and texture fidelity. Finally, in (f), where all loss terms are equally weighted ($\lambda_{1}=1.0,\lambda_{2}=1.0,\lambda_{3}=1.0$), the restoration results achieve the best overall performance, closely resembling the ground truth in terms of global structure, fine details, and color consistency. The red boxes highlight the critical differences in the restored areas, demonstrating that models with stronger structural loss tend to preserve global contours better, while those with dominant perceptual loss emphasize finer details. The overall analysis suggests that carefully balancing different loss terms is crucial for achieving high-quality image restoration. Experimental results confirm that using the full loss combination ($\lambda_{1}=1.0,\lambda_{2}=1.0,\lambda_{3}=1.0$) leads to optimal PSNR, SSIM, and visual performance, validating the effectiveness of DCAF-GAN in historical landscape restoration.

### Qualitative comparison analysis

**Fig 6:**
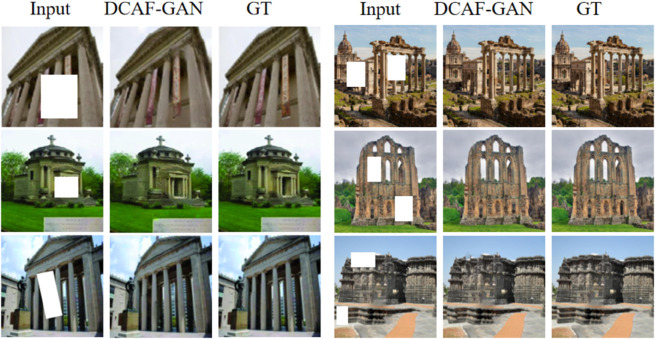
Visualization results of DCAF-GAN for historical landscape restoration. The figure compares the input corrupted images (Input), the restored images generated by DCAF-GAN (DCAF-GAN), and the corresponding ground truth images (GT).

The above Fig 6 presents the visualization analysis of DCAF-GAN in the task of historical landscape restoration. The figure includes the input corrupted images (Input), the restoration results generated by DCAF-GAN (DCAF-GAN), and the corresponding ground truth images (GT). From the comparison results, it is evident that DCAF-GAN can effectively restore the overall structure and fine details of various occluded regions. In the left-side examples, the input images contain significant occlusions, including both regular-shaped (rectangular missing parts) and irregular-shaped damages. The restoration results produced by DCAF-GAN exhibit high similarity to the ground truth in terms of color, texture, and architectural structure. Notably, the model successfully reconstructs high-frequency details, such as columns and walls, demonstrating strong generative capabilities. Additionally, the transitions between restored areas and surrounding regions are smooth, with no apparent boundary artifacts, indicating the model’s robust contextual awareness. The right-side examples further validate the generalization ability of DCAF-GAN across different historical architectural scenes. Even in cases with multiple occlusion blocks, the model effectively reconstructs building contours and fine details, ensuring high visual fidelity. However, in certain large missing regions, the restored details appear slightly blurred, which may result from limitations in global semantic reasoning. Overall, DCAF-GAN demonstrates its effectiveness in restoring historical landscapes across different types of damage. The model achieves superior performance in terms of texture preservation, structural consistency, and semantic coherence, highlighting its potential applications in heritage conservation and digital restoration.

### User perceptual quality comparison

The results from the user perceptual study are summarized in Table 5, which presents the average ratings of each model across various criteria, including clarity, color consistency, structural consistency, and visual appeal. The models compared include DCAF-GAN (ours), SGRGAN, ConvSRGAN, and EdgeConnect.

**Table 5:** User perceptual quality comparison (scores from 1 to 5).

Model	Clarity (Avg)	Color Consistency (Avg)	Structural Consistency (Avg)	Visual Appeal (Avg)	Overall Rating (Avg)
DCAF-GAN (Ours)	4.5	4.7	4.6	4.8	4.6
SGRGAN	4.0	4.1	4.2	4.3	4.2
ConvSRGAN	4.3	4.4	4.3	4.5	4.4
EdgeConnect	3.9	4.0	4.1	4.2	4.1

The results indicate that DCAF-GAN outperforms the other models across all evaluation metrics. Specifically, DCAF-GAN received the highest ratings in clarity (4.5), color consistency (4.7), and visual appeal (4.8). These results suggest that DCAF-GAN produces restored images that are perceived as clearer, more consistent in color, and more visually appealing compared to the other methods. In contrast, SGRGAN and EdgeConnect received lower scores in terms of clarity and visual appeal, with SGRGAN having particularly poor performance in these areas. ConvSRGAN performed well overall, particularly in color consistency (4.4) and visual appeal (4.5), but still lagged behind DCAF-GAN in most categories. Overall, DCAF-GAN achieved the highest overall rating (4.6), demonstrating its superior performance in restoring historical landscape images. The subjective ratings from the user study align with the objective evaluation metrics, confirming DCAF-GAN’s strong capabilities in producing high-quality, visually consistent restorations.

## Discussion

In this study, DCAF-GAN has demonstrated its effectiveness in historical landscape restoration, successfully restoring both details and structure, particularly in texture restoration and maintaining global structural integrity. However, despite achieving satisfactory results, there are limitations. Firstly, when faced with large-scale damage or complex occlusions, restoration quality tends to decrease. Although multiple loss functions optimize image details, some blurry or artifact-prone regions still appear, particularly with multi-layered structures and complex scenes. These issues are particularly noticeable when handling large missing regions or intricate structures, where the model struggles to preserve fine details and global consistency. Secondly, while the model excels in texture recovery, style and color tone consistency with the real images could still be improved, as some discrepancies in details remain in certain scenes, especially when the original image has intricate patterns or nuanced color variations.

For future work, improving the model’s ability to handle large-scale missing regions is a key priority. Introducing more contextual information or multi-scale feature extraction networks could enhance the model’s understanding of global structure, especially in repairing large occluded areas. Additionally, optimizing the model’s handling of complex occlusions, particularly in multi-layered historical structures, is crucial. Further optimization of the loss function is also necessary to improve model performance. Incorporating more domain-specific knowledge or exploring self-supervised learning could enhance generalization to unseen data, especially from specific historical contexts. The model’s adaptability across different historical landscape scenes, especially complex structures, also requires improvement. One direction for this is the incorporation of multimodal learning methods based on image generation models to increase diversity and restoration capability. Furthermore, combining AI with traditional computer vision techniques, such as depth image restoration and 3D reconstruction, could offer breakthroughs in future restoration tasks. Lastly, the model’s ability to recover large-scale occlusions and handle intricate damage patterns will be explored further through advanced learning techniques and improved handling of semantic information. Finally, optimizing model architecture and reducing computational resource consumption remains a focus to improve training efficiency and extend the model’s applicability.

## Conclusion

In this study, we introduced a novel approach for historical landscape restoration, demonstrating significant improvements over existing methods. DCAF-GAN effectively restores both fine details and global structures, achieving superior results in key metrics such as PSNR, SSIM, LPIPS, and FID. The model’s success can be attributed to its dual-branch encoder and channel attention-guided fusion module, which enhance the restoration process. Although DCAF-GAN performs well on various types of damage, future work will focus on handling large occlusions and more complex scenarios. Overall, DCAF-GAN provides a powerful tool for cultural heritage preservation and digital restoration, with potential for further advancements in future research.
